# Urinary Oxidative Stress Biomarkers in Workers of a Titanium Dioxide Based Pigment Production Plant

**DOI:** 10.3390/ijerph17239085

**Published:** 2020-12-05

**Authors:** Flavia Buonaurio, Maria Luisa Astolfi, Silvia Canepari, Marco Di Basilio, Rocco Gibilras, Marco Mecchia, Maddalena Papacchini, Enrico Paci, Daniela Pigini, Giovanna Tranfo

**Affiliations:** 1 Department of Chemistry, Sapienza University, P.le Aldo Moro 5, 00185 Rome, Italy; flavia.buonaurio@uniroma1.it (F.B.); marialuisa.astolfi@uniroma1.it (M.L.A.); silvia.canepari@uniroma1.it (S.C.); 2 Department of Technological Innovations and Safety of Plants, INAIL, Products and Anthropic Settlements, Via di Fontana Candida 1, Monte Porzio Catone, 00078 Rome, Italy; m.dibasilio@inail.it (M.D.B.); r.gibilras@inail.it (R.G.); m.papacchini@inail.it (M.P.); 3 Department for Risks Assessment and Prevention, INAIL, Central Office, Via R. Ferruzzi 40, 00143 Rome, Italy; m.mecchia@inail.it; 4 Department of Occupational and Environmental Medicine, Epidemiology and Hygiene, INAIL, Via di Fontana Candida 1, Monte Porzio Catone, 00078 Rome, Italy; e.paci@inail.it (E.P.); d.pigini@inail.it (D.P.)

**Keywords:** urinary Titanium, ICP-MS, HPLC-MS/MS, occupational exposure, oxidative stress biomarkers

## Abstract

Titanium dioxide is produced or imported into the EU for over one million tons/year. The International Agency for Research on Cancer (IARC) classification is 2B, a possible inhalation carcinogen for humans. This study evaluates urinary biomarkers of oxidative stress in workers of a plant producing TiO_2_ pigment powder, having 0.25 µm average particle size and an ultrafine fraction, compared to unexposed subjects. Urine samples were collected from forty workers before and after the shift, from six employees of the same company and eighteen volunteers from the same geographical area. Titanium and other metals concentrations were measured by ICP-MS, while DNA, RNA, and protein oxidation products by HPLC/MS-MS. A statistically significant increase was found for the urinary concentration of Al, Cd, Cr, Cu, Fe, Mn, Pb, Ti, and Zr, and for all biomarkers of oxidative stress in post-shift workers’ urine samples. Urinary concentrations after the working shift were higher than for employees and volunteers pooled together for Cd, Mn, and Zr, and for the oxidative stress biomarkers 8-oxoGuo, 8-oxodGuo, and 3NO_2_Tyr. Biomonitoring studies on dose and effect biomarkers for TiO_2_ occupational exposure provide information useful for protecting workers’ health even in conditions that comply with health and safety standards, highlighting reversible effects of chronic exposure at very low doses.

## 1. Introduction

Titanium dioxide (TiO_2_) is a mineral often referred to as an “accessory mineral”, as a small percentage is present in soils and sediments [1]. The polymorphs of TiO_2_ rutile, anatase, and brookite, are all quite commonly found, since Titanium is the ninth most frequently found element on the earth’s crust. Rutile (CAS 1317-80-2) is the most stable phase [2,3], and it occurs especially in lower metamorphic grade rocks, such as slate; this fact helps to explain why crystals of the mineral are found in different geological environments.

Ilmenite (iron and Titanium oxide) is the most abundant Titanium-containing mineral with a TiO_2_ content between 35% and 65% [1]. This commonly occurs as a result of enrichment, in sedimentary deposits, during the weathering and transport processes.

TiO_2_ substance is manufactured and/or imported in the European Economic Area in more than 1,000,000 tons per year. It has a wide range of applications as a pigment to provide whiteness and opacity to products, such as paints, coatings, plastics, papers, inks, foods (as E171), pills and tablets, toothpaste, and cosmetics, where nanosized Titanium dioxide is used as physical sunscreens because of its strong UV light absorption capacities and its resistance to ultraviolet light.

In the studied manufacturing facility, TiO_2_ pigment is produced from ilmenite mineral, enriched with naturally occurring rutile, to form the rutile crystal. The sulfate process transforms these components into soluble sulfates using a reaction with sulfuric acid. The resulting mass is dissolved in water, decanted, filtered, and heated. Precipitation of TiO_2_, in the form of a hydrated gel, is obtained by further dissolving this solution. The gel is then calcined, and the particles obtained undergo a coating treatment using a variety of chemicals (zirconium sulfate, sodium aluminate, and aluminum sulfate). Further washing and drying processes precede a final grinding with a fluid jet of steam that transforms the material into a very fine white powder that is packaged in 25 kg paper bags or large bags of up to 1 ton.

Our study focused on rutile dust, i.e., the only type of product that has been produced at the plant for the past seven years.

For the occupational exposure to TiO_2_ of the plant workers, the applicable limits are those for inhalable dusts, of 10 mg/m^3^ and for the respirable dusts, of 3 mg/m^3^, as threshold limit value-time weighted average (TLV-TWA), applied to conventional 8-h working day, over 40 h per working week.

NIOSH recommends that the exposure limit for fine particles of TiO_2_ be set at 2.4 mg/m^3^, while for ultrafine TiO_2_ an exposure limit of 0.3 mg/m^3^ is set at, as time-weighted average concentrations up to 10 h a day for 40-h per working week [4].

Titanium dioxide dust, when inhaled, has been classified by the International Agency for Research on Cancer (IARC) as a Group 2B carcinogen, meaning that it is possibly carcinogenic to humans [5].

According to the classification provided by companies to the European Chemicals Agency (ECHA) according to the REACH (Registration, Evaluation, Authorization and Restriction of Chemicals) regulation, only the nanoform is suspected of causing cancer, while for the other forms, no hazards have been classified.

The ECHA guidance on safe use of titanium dioxide suggests observing good industrial hygiene practice for chemical handling, avoiding raising, and breathing dust [6]

Most studies performed in humans or animals at the skin level showed that nano-TiO_2_ does not penetrate beyond the outer layers of stratum corneum to viable cells and does not reach the general circulation, either in healthy or in compromised skin. The Scientific Committee on Consumer Safety (SCCS) concludes in no evidence of carcinogenicity (supported by the European Chemicals Agency), mutagenicity or reproductive toxicity after dermal exposure to nano-TiO_2_. According to the SCCS, nano-TiO_2_ from sunscreens poses no health risk when applied to the skin at a concentration up to 25%. In 2016, the EU Cosmetic Regulation made nano-TiO_2_ an authorized UV filter, except in spray products that could lead to lung exposure.

Titanium dioxide has very poor water solubility, and therefore, it has been considered an inert powder for many years. However, data showing rapid distribution, slow or ineffective elimination, and potential long term tissue accumulation are particularly important for the human risk assessment of ultrafine TiO_2_ inhalation [7]. After oral exposure, nano-TiO_2_ absorption and toxicity are limited [8].

Tissue distribution in rats that were given TiO_2_ nanoparticles orally for 13 weeks (7 days/week) showed that TiO_2_ nanoparticles were not significantly increased in sampled organs. Ti concentrations were not significantly increased in urine, while very high concentrations of Ti were detected in feces [9].

Workers handling Titanium dioxide, especially in nanoparticles, and other metal oxides, showed increased levels of oxidative stress markers [10]. Oxidative stress, which causes cells to fail in maintaining normal physiological redox-regulated functions, leads to DNA damage, epigenetic changes, protein and lipid alterations, unregulated cell signaling, change in cell motility, cytotoxicity, apoptosis, and the onset of cancer.

ROS attacks on DNA and RNA lead to urinary elimination of 8-oxo-7,8-dihydroguanine (8-oxoGua), 8-oxo-7,8-dihydro-2′-deoxyguanosine (8-oxodGuo), and 8-oxo-7,8-dihydroguanosine (8-oxoGuo), which are considered biomarkers of oxidatively generated damage of DNA and RNA.

The methylation product of cytidine, 5-methylcytidine (5-MeCyt), has also been quantified as an epigenetic marker of RNA, and reduced levels of this modified nucleoside have been found to be associated with various tumors [11]. This oxidation process can also involve lipids and proteins: For proteins, it implies the introduction of functional groups that could alter their functionality and metabolism. One of the most important biomarker of protein oxidation is 3-Nitrotyrosine (3-NO_2_Tyr), an oxidation product of tyrosine, an amino acid found in most proteins, produced in the reaction with NO or NO_3_ [12]. Local biological effects in the respiratory system and increased levels of oxidative products of nucleic acids and proteins were found in the exhaled breath condensate of the (nano)TiO_2_ workers in a study on 36 male workers examined over 2012 and 2013, and this effect was lower in less exposed workers [13].

8-OxodG levels and antioxidant enzyme activities were also assessed in 227 workers, including 32 nano-TiO_2_ workers, 39 nano-SiO_2_ workers, 56 Indium-Tin oxide workers, showing that exposure to TiO_2_, SiO_2_, and ITO resulted in significant lower antioxidant enzymes (glutathione peroxidase and superoxide dismutase) and higher oxidative biomarkers 8-hydroxydeoxyguanosine (8-oxodG) than in 100 non-exposed controls [10].

Human biological monitoring (HBM) has been acknowledged as a useful tool to assess occupational and environmental exposure to chemicals. For those pollutants that are present both in the environment and in the workplaces, the values found in the general population provide the reference value (RV) for the interpretation of the data measured in the workers [14].

Urinary Titanium is not considered a suitable occupational dose biomarker, probably because inhalation has been judged a negligible source if compared to oral exposure sources, mainly food. Few papers report about the levels of Titanium in the human urine in the scientific literature [15,16,17,18,19,20,21].

The objective of this paper is to evaluate some urinary biomarkers of nucleic acids and protein oxidation in workers of a plant producing TiO_2_ pigment powder, having 0.25 µm average particle size, with an ultrafine/nanometric fraction, compared to those of unexposed subjects. The determination of urinary Titanium and some other elements, which can be found as impurities or in the coating of TiO_2_ powder, was also performed in all subjects to understand if the fine and ultrafine particles inhaled could be, differently from those ingested, absorbed, and excreted in the urine of the workers.

## 2. Materials and Methods

### 2.1. Information on the TiO_2_ Product

The characterization of the final product is not an objective of this paper, but some information is needed for the correlation with the occupational exposure assessment and with the results of biomonitoring.

Samples of the rutile TiO_2_ powder were characterized by X-Ray Diffraction analysis (Xrd), using a Philips Analytical diffractometer, and by Dynamic Light Scattering (DLS) analysis using a Zetasizer nano ZS (Malvern, UK).

From the results of the X-Ray diffractometry, we can deduce that the product consists of almost pure rutile mineral (about 99%).

In relation to the size of the TiO_2_ material, many studies demonstrate that light scattering is maximized in particles that are 0.2–0.3 μm in diameter, and most commercial products used as pigments have modal primary particle sizes within this range [5]. Therefore, a mean pigment size of 250 nm is what the Titanium dioxide makers are usually producing [22].

As for the DLS analysis, these measurements were performed on three different concentrations: 2, 20, and 200 µg/mL, and yielded particle sizes (diameters) ranging from 100 to 1000 nm.

According to the company laboratory results, the average crystal size of the finished product is approximately 240 nm, while coated particles measure about 300 nm. Coating additives, which vary in relation to the finished product, do not exceed 9% SiO_2_, 4.8% Al_2_O_3_, and 0.4% P_2_O_5_, or up to 0.03% ZrO_2_.

### 2.2. Studied Population

The studied group included forty workers, occupationally exposed to TiO_2_ dust, six employees of the same company, and eighteen volunteers residing in the same geographical area. Each subject was asked to complete a questionnaire to collect information on age, lifestyle, smoking, drug use, working activities, hobbies, and use of chemicals. Before providing the urine sample, everyone gave written informed consent to participate in the study.

The manufacturing company reported an average level of inhalation exposure for TiO_2_, respirable and inhalable dusts in its risk assessment document. exposure to this substance is below the TWA threshold limits established by the American Conference of Governmental Industrial Hygienists (ACGIH). Specific analytical tests are periodically carried out for Respirable and Inhalable dusts using personal or environmental samplers, particularly for workers engaged in industrial cleaning and maintenance operations, to confirm this assessment.

The study was a non-interventional/observational study based on definitions of the European Directive 2001/20/EC, for which the approval of an ethics committee is not required [23]; it was conducted according to the declaration of Helsinki and followed the International Code of Ethics for Occupational Health Professionals [24] published by the ICOH (International Committee of Occupational Health) and also shared by INAIL. Collected information was used as aggregate health data referred to the whole group of workers, with no possibility of individual identification. All subjects signed informed consent along with the questionnaire.

In Table 1, the main subjects’ characteristics are reported.

### 2.3. Urine Samples Collection

Urine samples were collected in sterile plastic containers by the subjects, immediately divided into three aliquots in polypropylene screw-cap tubes, and then transported refrigerated to the laboratory where they were stored frozen at −20 °C until analysis.

One aliquot of each urine sample was used to determine urinary oxidative stress biomarkers, one for the creatinine concentration and the third for the determination of Titanium and other elements. The final concentration of the analytes was expressed in µg/g of creatinine to normalize their values with respect to the variability of urine dilution.

Urinary creatinine was determined by the method of Jaffè using alkaline picrate test with UV/Vis detection at 490 nm [25]: Samples having a creatinine concentration higher than 3 g/L or lower than 0.3 g/L were discarded in accordance with the ACGIH recommendations [26].

### 2.4. Chemicals and Supplies

The analytical reference standards of 8-oxoGua, 8-oxoGuo, and 8-oxodGuo were purchased from Spectra 2000 s.r.l (Rome, Italy). The isotope-labeled internal standard (13C15N2) 8-oxoGua (98%) was obtained from Cambridge Isotope Laboratories Inc. (Tewksbury, MA, USA), (13C15N2) 8-oxoGuo (13C15N2) and 8-oxodGuo were obtained from CDN Isotopes Inc. (Pointe-Claire, QC, Canada). 3-NO_2_Tyr was purchased from Cayman Chemical Company (Ann Arbor, MI, USA) and 3NO_2_Tyr d3 from TRC (Toronto, ON, Canada). Glacial acetic acid 30% NH_3_, dimethyl sulfoxide, sodium hydroxide solution (50–52% in water), CHROMASOLV^®^ gradient grade 99.9% methanol and acetonitrile for HPLC/MS 99.9%, and low benzene content carbon disulfide were obtained from Sigma Aldrich (Saint Louis, MO, USA). Purified water was obtained from a Milli-Q Plus system (Millipore Milford, MA, USA). Anotop 10LC syringe filter devices (0.2 m pore size, 10 mm diameter) were purchased from Whatman Inc. (Maidstone, UK). Luna 5 µ C8 100 Å (250 × 4.6 mm) (Phenomenex, Torrance, CA, USA) and Discovery C18 (150 × 4.6 mm, 5 µm) (Supelco Analytical, Bellefonte, PA, USA) were used throughout the study.

### 2.5. Analytical Determination of Urinary Elements

The content of 12 elements (Al, As, Cd, Cr, Cu, Fe, Mn, Pb, Si, Ti, Zn, and Zr) in urine samples was determined by inductively coupled plasma mass spectrometry (ICP-MS; 820-MS; Bruker, Bremen, Germany) equipped with a collision reaction interface (CRI), as described previously with minor modifications [17,27,28]. Therefore, Al, Cd, Cu, Pb, Si, Zn, and Zr were monitored in standard mode, while As, Cr, Fe, and Mn were determined by CRI with He and H_2_ (99.9995% purity; SOL Spa, Monza, Italy) as cell gases, and Ti was quantified by CRI with He as cell gas. The operating conditions and parameters of ICP-MS have been previously detailed elsewhere [29]. Urine samples were ten times diluted with 2% HNO_3_ (*v*/*v*) (assay >67%; Promochem, LGC Standards GmbH Wesel, Germany) in polypropylene tubes (Artiglass s.r.l, Due Carrare, PD, Italy) and filtered using a 0.45 µm pore size syringe filter (GVS Filter Technology, Indianapolis, IN, USA). Method detection limits (MDLs) were determined as three times the standard deviation of blank determination (ten replicates). The blanks were prepared in the same way as the samples, but without adding urine. The MDLs for all the studied elements are the following: Al, 6 mg/L; As, 10 mg/L, Cd, 2 mg/L; Cr, 3 mg/L; Cu, 25 mg/L; Fe, 20 mg/L; Mn, 2 mg/L; Pb, 0.7 mg/L; Si, 150 mg/L; Ti, 2 mg/L; Zn, 20 mg/L, and Zr, 0.03 mg/L.

### 2.6. Analytical Determination of Urinary Oxidative Stress Biomarkers

All the urine samples were analyzed by liquid chromatography/tandem mass spectrometry (HPLC/MS-MS) on an API 4000 triple-quadrupole mass spectrometry detector equipped with a Turbo Ion Spray (TIS) probe (AB Sciex, Framingham, MA, USA) coupled to a Series 200 LC quaternary pump (PerkinElmer, Norwalk, CT, USA).

Detection was in the MRM mode, and parameters were optimized for the analytes by the automated “infusion quantitative optimization” procedure and subsequently refined by flow injection analysis (FIA) using the pure standards.

The concentration of 8-oxoGua, 8-oxoGuo, 8-oxodGuo, and 3-NO_2_tyrosine was determined following the method described by Andreoli et al. [30], with some modifications in the sample thawing, dilution solvents, chromatographic column, and mobile phases. Moreover, for 5-methylCytidine (5-MeCyt) the method of Andreoli et al. [30] was followed, after diluting the sample 1:100 and using a different chromatographic column; Cotinine d3 was used as the internal standard for 5-MeCyt. Before the analysis, samples were thawed in lukewarm water at about 37 °C, vortexed, centrifuged at 10.000× *g* for 5 min; the urine supernatant was added to the internal standard and injected into the HPLC-MS/MS system. The precursor/product ionic transitions monitored (positive ion mode) were 168.0→140.0 and 171.0→143.0 for 8-oxoGua and its internal standard ((13C15N2) 8-oxoGua), 284.3→168.0 and 287.13→171.1 for 8-oxodGuo and its internal standard ((13C15N2) 8-oxodGuo), 300.24→168.2 and 303.24→171.0 for 8-oxoGuo and its internal standard ((13C15N2) 8-oxoGuo), 226.99→181.0 and 229.99→184.0 for 3-NO_2_tyrosine and its internal standard (3-NO_2_tyrosine d3), 257.95→126.100 and 180.3→80.10 for 5-methylCytidine and Cotinine d3 used as internal standard, respectively. The 1.5 version of Analyst^®^ software (AB Sciex, Framingham, MA, USA) was employed for instrument control.

### 2.7. Statistics

Statistical analyses were performed using an additional program of Microsoft Office Excel (Microsoft Corporation, Redmond, WA, USA), Analysis Tool Pak, after checking the log-normality of the distribution of data. The T-test for independent variables was used to analyze the difference between log-transformed values of the urinary parameters measured before and after the work shift in workers and between exposed workers and non-exposed subjects. The linear bivariate correlation between variables was also determined on the log-transformed values. In all tests, a *p*-value lower than 0.05 was considered as statistically significant.

## 3. Results and Discussion

The analytes concentration levels found for the study subjects have been expressed in terms of mean with the standard deviation, 5th, 50th or median, and 95th percentile, in µg/g creatinine, and reported in Table 2 for the twelve measured elements, including Titanium, and in Table 3 for the five oxidative stress biomarkers.

In the workers’ urines, biomarkers have been measured before and after the work-shift, while for the non-exposed subjects, a spot urine sample was used.

The values found in the six employees of the same company have been presented separately, but due to their small number, these results have been pooled together with those of the 18 Volunteers to be compared to those of the workers.

Results show a statistically significant increase of the concentrations of some elements, namely, Al, Cd, Cr, Cu, Fe, Mn, Pb, Ti, and Zr, in the urine of the workers during the working shift. For what concerns the occupational biological exposure limits, the ACGIH stated a BEI in the urine only for Cd and Cr, respectively, equal to 5 µg/g creatinine (any time) and 10 µg/g creatinine as increase after the working shift [31], and the mean values measured for these elements are just below the limits.

However, if we compare the end of work-shift values of the workers with those of the 24 non-exposed subjects, only Cd, Mn, and Zr are still statistically different. This can be interpreted as a reversible increase of the considered elements, which are still within the range of the non-exposed subjects in the case of Al, Cr, Cu, Fe, Pb, and Ti. In the non-exposed subjects group, there were some very high urinary concentrations of As and Pb, but these values were not eliminated from the study.

In the case of Cd, the mean urinary level of the workers measured after the work-shift is higher than in the non-exposed group, but below the ACGIH occupational exposure limit.

For Mn, there is a poor relationship between external exposure and urine manganese concentration, as absorbed Manganese is eliminated with a half-life of 10 to 30 days [32]. For this reason, an occupational biological exposure limit was not established.

For Zr, the increase showed during the work-shift could be linked to its presence in the process, as it is one of the elements used for the particles coating; but few studies is present for this element in human urine: Less than 2 µg/L have been reported in the urine of 30 subjects in an Italian study of 1990 [33].

For what concerns the Titanium concentration in the urine, we must remember that Titanium dioxide has very poor water solubility and that the limited amount that reaches the urine should be dissolved to be detected. Therefore, the sample preparation method used has a crucial role in determining the amount of Titanium that can be found in the urine samples. In this work, our considerations are not based on the absolute values, but only on the comparison between samples treated with the same (or comparable) procedure for TiO_2_ dissolution (HNO_3_ treatment).

Levels of urinary Titanium of general population subjects are reported in comparable studies: 9.97 µg/g creatinine in 100 Viennese subjects [16], 5.87 µg/g creatinine in 132 subjects of UK population [18], 33.1 µg/g creatinine in 2004 residents of Wuhan (China) aged 29–75 years [21]. There are some published studies on children that were not considered in this comparison as children always present higher biomarkers values than adults.

The mean values of the urinary concentration of all the oxidative stress biomarkers found in the workers at the end of the work-shift are higher than before the work-shift, with a very high statistical significance (*p* < 0.01). A circadian rhythm of these biomarkers can be excluded because they showed to be not influenced by the time of the day based on the existing literature [34,35].

The urinary concentrations of 8-oxoGuo, 8-oxodGuo, and 3NO_2_Tyr measured in workers at the end of the working shift are higher than that of non-exposed subjects (employees and volunteers pooled together) and this difference is statistically significant (*p* < 0.05). For 5-MeCyt, the urinary concentration is lower in the exposed subjects than in the not-exposed (*p* < 0.01), indicating a statistically significant reduction associated with the occupational exposure status.

The absolute values found in the workers have also been compared to the values found in a comparative study carried out in the same laboratory [36].

The comparison is shown in Table 4.

Data show that the mean values of 8-oxoGua and 8-oxoGuo measured before the work-shift are in the range of the values found in the general population. The mean value of 8-oxodGuo found before the work-shift, and the end-shift mean values of all the three nucleic acid oxidative stress biomarkers are higher than those found in the general population and comparable to those of other groups occupationally exposed to chemicals (gasoline pump attendants and painters).

The correlation between the urinary levels of the studied elements and the concentrations of the oxidative stress biomarkers has also been studied both on the results of the 40 TiO_2_ workers at the end of the shift and on the complete dataset: Workers before their shift, workers after their shift, and non-exposed subjects, *n* = 104. The results are reported in Table 5.

In the pooled data, Al, Cd, Cr, Cu, Mn, Fe, Ti, and Zr are positively correlated with the oxidative stress biomarkers, in particular with 8-oxoGua and 3-NO_2_Tyr. A positive trend is also observed for 8-oxodGuo, suggesting a smaller association of these exposure levels with the DNA oxidative damage with respect to damage to RNA and proteins.

In the workers at the end of the shift (ES), significant positive correlations exist between the urinary levels of Cd, Cr, Cu, Fe, Mn, Pb, Ti, and Zr with four of the measured oxidative stress biomarkers, namely, 8-oxoGua, 8-oxoGuo, 5-MeCyt, and 3-NO_2_Tyr, showing that exposure to these metals is associated with RNA and protein oxidative damage.

Age or job seniority are correlated among them, but not with any of the measured biomarkers in the exposed subjects.

## 4. Conclusions

The results of this study show that occupational exposure to the process of TiO_2_ pigment powder production involves a reversible increase in the urinary concentration of some elements (Al, Cu, Zr, Pb, Cr, Mn, Fe, and Ti) and of the oxidative stress urinary biomarkers 8-oxoGua, 8-oxoGuo, 8-oxodGuo, and 3-NO_2_tyrosine.

However, the absolute levels of these biomarkers are still within the range of the general population levels, or, where existing, below the occupational biological limits, confirming the company risk assessment results.

With reference to the TiO_2_ urinary concentration, even if its absolute value is strongly dependent on the analytical method used, it appears to be sensitive to inhalation exposure. We can hypothesize that, as only the ultrafine/nanometric fraction of the dust inhaled can reach the body fluids and be excreted with the urine, the increase of urinary Ti concentration during the working shift could be considered linked to the occupational exposure to this fraction, which is the most active in terms of health effects.

This study confirms that the effect biomarkers for oxidative stress determined in this study can provide useful information for protecting the workers’ health, even in conditions of compliance with health and safety standards, highlighting the reversible effects of chronic exposure to very low doses of Titanium dioxide and other elements.

## Figures and Tables

**Table 1:** Characteristics of the subjects.

Subjects	*n*	Age (Years)	Company Seniority (Years)	Work Seniority in the Job (Years)	Average Working Hours/Five Days
Workers	40	25–60	2–30	1–30	39
Employees	6	36–63	2–39	2–18	38
Volunteers	18	22–66	-	-	-

**Table 2:** Urinary concentrations of Titanium and other elements in workers, before and after the working shift, and in not-exposed subjects (employees and volunteers).

µg/g Creatinine	Al	Si	Cu	Zn	Zr	Pb	Cr	Mn	Fe	As	Cd	Ti
	**Workers (Before work-shift) *n* = 40**											
Mean	31.11 *	7503.49	38.60 *	509.73	0.16 *	1.17 *	8.29 *	2.09 *	24.52 *	61.46	2.12 *	13.68 *
STD	77.65	2668.58	22.21	256.77	0.39	0.89	5.17	1.43	16.61	93.94	1.45	4.41
5° perc	4.16	4611.21	15.75	238.59	0.02	0.42	2.73	0.79	10.27	8.15	0.79	8.61
Median	12.31	6878.70	33.10	468.18	0.06	0.94	7.34	1.64	18.15	26.07	1.58	12.55
95° perc	79.22	10993.81	86.84	929.78	0.74	3.43	17.07	5.31	65.27	167.04	5.36	23.54
	**Workers (After work-shift) *n* = 40**											
Mean	81.36	6071.85	78.49	559.33	3.56	2.06	16.54	4.79	51.71	57.12	4.77	20.90
STD	176.45	2902.50	77.93	329.31	17.12	2.01	15.06	5.59	53.86	55.58	5.64	16.53
5° perc	4.50	2544.84	14.94	190.42	0.04	0.53	5.31	1.14	11.89	8.92	1.03	8.00
Median	25.11	5895.04	43.85	485.58	0.15	1.03	10.74	2.06	26.39	35.50	2.08	14.41
95° perc	299.73	9948.80	220.63	1390.76	3.85	6.06	49.26	17.52	167.06	173.15	17.67	61.66
	**Employees (*n* = 6)**											
Mean	116.94	5364.91	37.85	350.80	0.14	16.81	8.23	2.88	61.21	32.93	2.01	14.13
STD	211.95	1971.00	15.12	113.44	0.11	14.00	3.82	2.20	66.30	20.43	0.62	5.74
5° perc	6.73	3836.17	18.10	195.34	0.03	2.85	3.89	1.18	11.19	18.11	1.16	7.90
Median	22.03	4317.47	38.45	408.62	0.14	14.95	8.52	2.20	24.39	27.32	2.09	13.08
95° perc	432.59	7901.39	53.60	443.46	0.28	33.28	12.74	6.10	153.27	62.91	2.67	20.69
	**Not Exposed Subjects (*n* = 24)**											
Mean	54.05	7322.85	54.65	678.35	0.09 *	35.20	12.14	2.48 *	37.82	192.03	2.29 *	16.62
STD	112.51	3025.85	35.12	1196.62	0.08	46.31	7.84	1.82	38.31	350.16	1.53	6.46
5° perc	5.45	3807.89	24.30	198.77	0.01	3.26	3.69	0.93	9.79	18.02	0.94	8.68
Median	17.80	7074.62	42.40	419.79	0.06	23.54	10.19	1.88	21.42	65.66	1.88	15.88
95° perc	186.52	12343.95	115.19	814.17	0.23	96.96	25.94	6.32	124.37	1058.38	5.51	30.02

* Statistically significant difference with the corresponding value in workers after the working shift (*p* < 0.05).

**Table 3:** Urinary concentrations of biomarkers of nucleic acid oxidation and nitro-oxidation in workers, before and after the working shift, and in not-exposed subjects (employees and volunteers).

µg/g Creatinine	8-oxoGua	8-oxoGuo	8-oxodGuo	3-NO_2_Tyr	5-MeCyt
	**Workers (Before work-shift) *n* = 40**				
mean	32.21	11.53	14.31	12.60	0.86
STD	17.26	5.99	5.13	7.60	0.50
5° perc	11.18	5.21	6.77	5.31	0.33
median	32.11	11.08	14.30	9.35	0.74
95° perc	66.42	22.21	22.86	29.27	2.04
	**Workers (After work-shift) *n* = 40**				
mean	57.36	16.02 *	19.69 *	24.22 *	1.60 **
STD	55.78	9.64	14.01	24.21	1.72
5° perc	13.70	5.50	9.99	5.67	0.39
median	34.81	14.06	15.29	13.67	1.01
95° perc	171.08	33.15	48.79	78.05	4.45
	**Employees (*n* = 6)**				
mean	39.84	8.43	14.97	10.88	1.44
STD	30.43	3.70	4.68	3.58	0.47
5° perc	11.22	5.20	10.63	7.31	0.84
median	30.78	7.82	13.90	10.18	1.66
95° perc	83.32	13.66	21.68	15.89	1.84
	**Not Exposed Subjects (*n* = 24)**				
mean	41.35	8.89 *	14.66 *	11.93 *	1.95 **
STD	30.97	3.88	6.73	7.12	1.15
5° perc	8.67	5.50	6.31	5.35	0.84
median	31.36	7.65	13.30	9.42	1.60
95° perc	100.50	15.79	26.88	27.74	4.06

Workers after the work-shift versus non-exposed subjects: * *p* < 0.05; ** *p* < 0.01.

**Table 4:** Nucleic acids Oxidative stress levels in workers and general population subjects.

Groups	Gasoline Pump Attendants (Saudi Arabia) *n* = 29	Gasoline Pump Attendants (Italy) *n* = 102	Fiberglass Workers (Italy) *n* = 24	Painters (Italy) *n* = 17		Divers (Exposed to Hyperbaric Atmosphere) (Italy) *n* = 6		General Population (Italy) *N* =132	Titanium Workers (Italy—This Study) *N* = 40	
				Before Shift	End Shift	Before Shift	End Shift		Before Shift	End Shift
	**8-oxoGua (µg/g creatinine)**									
Mean	55.92	81.83	100.89	13.83	11.20	25.60	27.18	36.29	32.21	57.36
(SD)	(63.61)	(60.99)	(78.82)	(15.41)	(13.69)	(14.06)	(4.78)	(35.81)	(17.26)	(55.78)
	**8-oxoGuo (µg/g creatinine)**									
Mean	29.16	10.63	34.00	10.27	16.13	21.45	36.18	12.19	11.53	16.02
(SD)	(15.70)	(5.54)	(9.95)	(6.87)	(6.12)	(6.59)	(8.64)	(5.56)	(5.99)	(9.64)
	**8-oxodGuo (µg/g creatinine)**									
Mean	15.15	4.07	27.80	2.86	5.53	3.01	5.97	7.83	14.31	19.69
(SD)	(9.10)	(1.69)	(11.82)	(0.89)	(1.90)	(0.78)	(1.20)	(3.47)	(5.13)	(14.01)

**Table 5:** Pearson’s correlations between elements and oxidative stress biomarkers in pooled data and in workers at the end of the shift.

Biomarker	Al	Cu	Zr	Pb	Cr	Mn	Fe	Cd	Ti
8-oxoGua	0.51	0.77	0.53	0.21	0.62	0.84	0.72	0.82	0.73
8-oxoGuo	0.39	0.50	0.51	−0.07	0.47	0.64	0.45	0.66	0.49
8-oxodGuo	0.25	0.21	0.33	−0.05	0.23	0.30	0.19	0.29	0.23
3-NO_2_Tyr	0.53	0.82	0.61	0.14	0.72	0.91	0.77	0.94	0.77
5-MeCyt	0.46	0.79	0.37	0.52	0.61	0.78	0.73	0.78	0.69
8-oxoGua_ES	0.57	0.82	0.55	0.83	0.77	0.90	0.79	0.92	0.81
8-oxoGuo_ES	0.46	0.55	0.50	0.66	0.55	0.70	0.51	0.72	0.56
8-oxodGuo_ES	0.31	0.09	0.32	0.19	0.16	0.18	0.05	0.19	0.06
3-NO_2_Tyr_ES	0.63	0.87	0.61	0.85	0.81	0.95	0.83	0.96	0.85
5-MeCyt_ES	0.56	0.84	0.53	0.77	0.71	0.89	0.83	0.89	0.76
